# Supplementary material for: Global Investigation of Cytochrome P450 Genes in the Chicken Genome
Source: Genes (Basel). 2019 Aug 14;10(8):617. doi: 10.3390/genes10080617 (PMC6723978; doi:10.3390/genes10080617)
Supplement: Supplementary file 1 [file genes-10-00617-s001.zip › Table S1.docx]

Table S1 List of primers used in the study

| **Gene name** | **Gene ID** | **Sequence (5’ to 3’)** | **Product size（bp）** |
| --- | --- | --- | --- |
| cCYP2C23a | ENSGALG00000005795 | F: CCCAAGTGGGTGACTGCATA  R: CACACCCTGAAACATCGGGA | 115 |
| cCYP4B7 | ENSGALG00000010469 | F: AGGTGACACGGAGCTACTCT  R: TCTCTGGTCCCCAAGGTAGG | 188 |
| cCYP4V2 | ENSGALG00000013535 | F: CTGGTTAGGTCCACTGCCTG  R: GCCATGGGTGCAGAAATGTG | 116 |
| cCYP2AC1 | ENSGALG00000016690 | F: GGAAGTCCATCAGTGTCGCT  R: CGTCGATGAAGCTCCTCTGG | 169 |
| cCYP4A22 | ENSGALG00000020688 | F: ACCCTATCTGGTTTGGGGGT  R: CAGGAGCTTCCGATGTTGGT | 182 |
| cCYP2W1 | ENSGALG00000021238 | F: TAATGGGTCTGCTGTGTGCAG  R: ACGCTGCATTTTTAGAGCCG | 72 |
| cCYP2C45 | ENSGALG00000023925 | F: GATCGTGCGGATGAGTTTGC  R: AACCATCCCTCGTTGTTGCT | 98 |
| cCYP2B4L | ENSGALG00000038694 | F: GAGATTTCATCAGCGCCTTC  R: ACCACATCCAACTCCTCCTG | 208 |
| cCYP2AC2 | ENSGALG00000046639 | F: CCAAGCACAACACCCTTCAC  R: CCAATGATGGGTAACGCCCT | 188 |
| cCYP51A1 | ENSGALG00000009365 | F: ATACAGGAGCAGTGCTACGC  R: GTTGTATCCTGCGACGGTCT | 186 |
| cCYP7A1 | ENSGALG00000041222 | F: GTAACGCCCTAGATGCCCTC  R: GCTCTCTCTGTTTCCCGCTT | 220 |
| cCYP1A1 | ENSGALG00000001325 | F: GAGCTGGATCAGACCATCGG  R: GCTTGAAGGAAGGAGGGTCC | 250 |
| cCYP1A2 | ENSGALG00000001320 | F: GTACACCACGCTTCCCCTTA  R: CTCCATCACGTCCCCGTATT | 289 |
| cCYP3A4 | ENSGALG00000004436 | F: TTTTGCAACGAGGCATTGGG  R: TCCGAGGCCAAGGAATTGTC | 78 |
| cCYP2D6 | ENSGALG00000011894 | F: TGGAACCCTGCTTACATCCG  R: ATGACGGCATTGGTGTAGGG | 293 |
| cCYP4F11 | ENSGALG00000039257 | F: TCTGTCCGACGAGGACATCT  R: GGCATTGCTCCTGGTAGTGT | 128 |
| APOV1 | ENSGALG00000015134 | F: CAATGAAACGGCTAGACTCA  R: AACACCGACTTTTCTTCCAA | 108 |
| β-actin | ENSGALG00000009621 | F: GAGAGAAGATGACACAGATC  R: GTCCATCACAATACCAGTGG | 116 |
